# Bio-Inspired Intelligent Systems: Negotiations between Minimum Manifest Task Entropy and Maximum Latent System Entropy in Changing Environments

**DOI:** 10.3390/e25111541

**Published:** 2023-11-14

**Authors:** Stephen Fox, Tapio Heikkilä, Eric Halbach, Samuli Soutukorva

**Affiliations:** VTT Technical Research Centre of Finland, FI-02150 Espoo, Finland; tapio.heikkila@vtt.fi (T.H.); eric.halbach@vtt.fi (E.H.); samuli.soutukorva@vtt.fi (S.S.)

**Keywords:** adaptability, automated negotiation, bio-inspired, intelligent systems, latent system entropy, manifest task entropy, robotics, stability

## Abstract

In theoretical physics and theoretical neuroscience, increased intelligence is associated with increased entropy, which entails potential access to an increased number of states that could facilitate adaptive behavior. Potential to access a larger number of states is a latent entropy as it refers to the number of states that could possibly be accessed, and it is also recognized that functioning needs to be efficient through minimization of manifest entropy. For example, in theoretical physics, the importance of efficiency is recognized through the observation that nature is thrifty in all its actions and through the principle of least action. In this paper, system intelligence is explained as capability to maintain internal stability while adapting to changing environments by minimizing manifest task entropy while maximizing latent system entropy. In addition, it is explained how automated negotiation relates to balancing adaptability and stability; and a mathematical negotiation model is presented that enables balancing of latent system entropy and manifest task entropy in intelligent systems. Furthermore, this first principles analysis of system intelligence is related to everyday challenges in production systems through multiple simulations of the negotiation model. The results indicate that manifest task entropy is minimized when maximization of latent system entropy is used as the criterion for task allocation in the simulated production scenarios.

## 1. Introduction

Scientific research has revealed some fundamental principles that underlie the operation of nature. In natural sciences, it is recognized that survival and growth depend on balancing adaptability with stability [1,2,3,4,5,6]. In physics and in neuroscience, higher adaptability is associated with higher intelligence, which is described in terms of entropy. In theoretical physics, increased intelligence is associated with increased entropy, which entails potential access to an increased number of states that could facilitate adaptive behavior [7]. Similarly, in theoretical neuroscience, it is argued that there is a positive relationship between neural entropy and human intelligence. Specifically, potential access to a higher number of neural states is associated with higher intelligence. This is because potential access to a higher number of neural states could better enable predictions about the environment and so better enable adaptive behavior [8]. However, potential to access a larger number of states is a latent entropy, as it refers to an indeterminate number of neural states that could possibly be accessed, and it is also recognized that neurological functioning needs to be efficient [9] through the minimization of manifest entropy [10]. Similarly, in theoretical physics, the importance of efficiency is recognized through the observation that nature is thrifty in all its actions [11]. Moreover, it is argued that survival and growth depend upon evolution towards work being carried out with least action [12], this being necessary in order to counteract locally the tendency towards maximum entropy universally. Thus, intelligence needs to encompass balance between adaptability, facilitated by maximum latent entropy, and stability facilitated by minimum manifest entropy [13].

In this paper, findings are reported from research that has investigated the potential for balancing adaptability and stability in production organizations by maximizing latent system entropy and minimizing manifest task entropy. Findings are reported in the remaining six sections. In Section 2, a review is provided of previous literature concerned with bio-inspired production systems. In Section 3, a more detailed analysis is provided of the interplay between latent system entropy and manifest task entropy in balancing adaptability and stability. This analysis is related to different types of production work. In Section 4, it is explained how automated negotiation can enable the balancing of adaptability and stability. In Section 5, a mathematical negotiation model for balancing latent system entropy and manifest task entropy is presented. In Section 6, the first principles analysis of system intelligence is related to everyday challenges in production systems through multiple simulations of the negotiation model. In conclusion, in Section 7, principal contributions are stated, practical implications are discussed, and directions for future research are proposed. Readers who are not particularly interested in industrial applications could skip Section 2, Section 3 and Section 4.

Overall, this paper goes beyond the previous literature relating insights from nature to intelligent production [14,15,16,17,18,19,20,21,22], which has not addressed the interplay between adaptability and stability in terms of latent system entropy and manifest task entropy. In particular, this paper introduces a new perspective of how first principles can be applied in systems that encompass natural and artificial intelligence. This new perspective goes beyond the state-of-the-art of formulations based on first principles and entropy dynamics [23,24,25,26].

## 2. State of the Art

Seeking insights from nature to improve production systems is well-established. For example, in the 1990s, it was proposed that the brain could provide a metaphor to model flexible production systems [27]. More broadly, in the following decade, it was argued that biological systems could provide useful analogies for manufacturing systems [28]. In 2016, there was a comprehensive review of biomimetics in production organizations, which revealed continuing interest in improving production systems through reference to biological systems [29]. Subsequently, in 2018, biologicalisation in manufacturing was proposed [14]. That is “use and integration of biological and bio-inspired principles, materials, functions, structures and resources for intelligent and sustainable manufacturing technologies and systems with the aim of achieving their full potential” [14].

It has been proposed that biologicalisation in manufacturing can have three development modes: inspiration, integration, and interaction [15]. Inspiration involves the translation of evolutionary biological phenomena into technical systems; integration involves the combination of biological systems and technical systems; and interaction involves self-sustaining biointelligent systems encompassing the technical, informational, and biological [15]. Although there is some recognition of nature’s balancing of adaptability and stability in the biologicalisation literature [16], this topic has not been the focus of previous studies, nor has the interplay between latent system entropy and manifest task entropy been considered. Rather, valuable work has been done in order to integrate various technologies in biointelligent systems [17,18], such as digital twins [19], selective laser melting [20], and intelligent machines and components [21]. In addition, work has addressed some aspects of the design of biointelligent systems [22].

An entropy-based first principles formulation that has been related to intelligent systems and intelligent robotics is the free energy principle (FEP) and its corollary, active inference theory (AIT). Within the FEP, living things seek to minimize the differences between what happens to them in the world and their internal model of themselves in the world (i.e., world model). Differences between predictions of what will happen based on the world model and what does happen are minimized through active inference. This inference combines perceptual inference, instrumental inference, and epistemic inference. Perceptual inference involves the inference of sensory stimuli from the world based on world models. This is summarized in idioms such as “we don’t see the world as it is but as we are” and “to a hammer everything looks like a nail”. Instrumental inference involves inference about what actions to take in the world to reduce differences between the world model and what is experienced in the world. Epistemic inference involves updating the world model to minimize the differences between the world model and what is experienced in the world [13]. FEP-AIT has been applied theoretically to designing intelligent systems [23], to deep learning [24], to robotics [25], and to interactions with robots [26]. However, the closer FEP-AIT has moved towards implementation examples, the farther away it has moved from the entropy-based first principles upon which it is based.

Meanwhile, balancing adaptability and stability remains a major challenge for manufacturing organizations. This balance can be described as organizational ambidexterity [30]: i.e., adaptability by one hand while stability by the other hand. In particular, adaptability is epitomized by engineer-to-order (ETO) companies that are open to producing whatever each individual customer may have in mind. By contrast, stability is epitomized by make-to-stock (MTS) companies that predetermine what they will produce in advance. ETO companies can attempt to increase their stability and MTS companies can attempt to increase their adaptability through mass customization. This involves predefinition of product subassemblies and their configuration options, and can be described as assemble-to-order (ATO). However, such initiatives are seldom entirely successful [31,32].

In practice, industrial software systems, such as product configurators, are set up to enable ATO. These are online brochures, which can enable potential end-users to select and configure sub-assemblies into their preferred products [33]. Although product configurators have been applied for several decades, their implementation remains challenging [34], and attempts to bring intelligence to product configurators are ongoing [35,36]. Furthermore, efforts to balance adaptability and stability by balancing production agility and production leanness in so-called leagile manufacturing continues to be challenging despite several decades of implementation efforts. Hence, there is ongoing research and development work in applying advanced technologies, including artificial intelligence, to improve leagile production [37,38,39]. However, as with the biologicalisation literature, this work has not encompassed balancing adaptability and stability through balancing of latent system entropy and manifest task entropy in manufacturing workforces comprising humans and robots. 

## 3. Balancing Latent Entropy and Manifest Entropy

As entropy is fundamental to behavior from particles [7] to communities [40], improving intelligent production systems through application of entropy constructs is within the scope of biologicalisation. Previous research by others has applied information-theoretic entropy in efforts to improve scheduling involving different levels of operational uncertainty [41]. This is appropriate because the information-theoretic entropy of a random variable can be defined as the average level of uncertainty inherent in the variable’s possible outcomes. Claude Shannon defined information-theoretic entropy in the 1940s [42]. This was preceded by definition of statistical physics entropy by Ludwig Boltzmann in the 1870s and Max Planck in the 1900s. Statistical physics entropy describes the disorder of a system. This entropy increases as the number of states available to the system increases [43]. Earlier in the 1860s, Rudolf Clausius described thermodynamic entropy based on observations that much energy in engines was lost due to dissipation and could not be converted into useful work. Thermodynamic entropy can be regarded as chaos in a thermodynamic system [44].

Manufacturing processes can involve interactions between information-theoretic entropy, statistical physics entropy, and thermodynamic entropy. An example of this is inadequate task instructions entailing the information-theoretic entropy of information uncertainty for production operatives. For example, if unclear instructions for a task entail information-theoretic entropy of 2.58 bits, there can be the statistical physics entropy of physical disorder due to there being six different equiprobable ways in which the task could be carried out. If only one of those six different ways of carrying out the work entails least action, there can be thermodynamic entropy from some of the production operatives’ potentially useful thermodynamic energy becoming useless thermodynamic energy as it is lost in five instances of inefficient work. An example of this would be if the assembly instructions for a cargo bike can be interpreted in six different ways, but five of them involve unproductive repeat handling of bike parts during assembly work. Accordingly, much of engineering is focused on continuous efforts to minimize information uncertainty to enable work to be carried out right-first-time with the least action [45].

The relationship between information-theoretic entropy and statistical mechanics entropy is straightforward. In particular, two to the power of the information-theoretic entropy indicates the statistical mechanics entropy. For example, two to the power of 2.58 is six. The thermodynamic entropy arising from statistical mechanics entropy is less straightforward, as it depends on factors such as mass, which can affect the amount of thermodynamic entropy lost in unproductive work due to physical disorder arising from information uncertainty. For example, the mass of a factory robot can be more than the mass of a human operative. Nonetheless, in terms of manifest task entropy, more information-theoretic entropy entails more physical disorder and more energy lost in unproductive work [46]. Hence, there is interest in minimizing manifest information-theoretic entropy in intelligent production systems [47,48].

However, the elimination of all entropy can lead to systems being too efficient for their own good because they cannot adapt to changes in the environment [49]. A system can become too stable and suffer from so-called oscillation death [50]. This involves the end of back-and-forth reciprocal exchanges of learning between the system and the environment. Back-and-forth reciprocal exchanges can be described in terms of rhythmogenesis, that is, rhythms being generated in many coupled physical, chemical, and biological systems in which feedback comes through underlying coupling [51].

Within manufacturing, coupling between companies and the environment is considered in terms of companies’ decoupling points for customer orders. In particular, the customer order decoupling point (CODP) is the point in the material flow where the product is tied to a specific customer order. The CODP is different for make-to-stock (MTS), assemble-to-order (ATO), make-to-order (MTO), and engineer-to-order (ETO). For MTS, the CODP is at the point of completed product, such as a customer selecting from alternative completed refrigerators, washing machines, etc., at a retail outlet. For ATO, the CODP is at the point of product assemblies and their configuration options, such as car body shapes and engine sizes. For MTO, CODP is at the point of product sub-assemblies, such as the sub-assemblies that can go into industrial machines for paper production. For ETO, CODP is at the level of formed materials and parts, such as the metal sheets and sections that go into the manufacture of one-of-a-kind ships [52,53].

The point at which the company is decoupled from the environment determines the extent of information uncertainty experienced by the company. For MTS, which has a CODP at the product level, there can be so little information uncertainty that production can be fully automated in so-called lights-out factories where there is no need for lighting or other facilities that are used when there are human factory operatives [54]. By contrast, for ETO, which has its CODP at the formed materials level, there can be information uncertainty about formed materials, parts, sub-assemblies, assemblies, and the final product. As a consequence, ETO production is characterized by high latent entropy arising from being open to whatever customers in the business environment may have in mind, and the by risk of high manifest task entropy due to the uncertainty that can arise from being open to whatever customers may have in mind. Accordingly, intelligent ETO production and intelligent MTO production needs to be able to adapt to whatever new customer orders may arise from the business environment, but do so while minimizing manifest task entropy [55,56].

The balancing of external adaptability and internal stability can be framed as an ongoing negotiation involving product descriptions and task descriptions. At the interface between the company and its customers in the business environment, negotiation can be carried out in terms of product descriptions, product price, and production durations. In simple terms, the more original the product description, the more customers have to pay and the longer customers have to wait [57]. The more open a manufacturing company is to making any product description that a customer can imagine, the more latent entropy from openness to the environment could manifest as manifest entropy within the manufacturing company. At the interface between the company’s sales department and the company’s production department, negotiation can be carried out in terms of workforce task descriptions derived from customer orders. In particular, the more open a manufacturing company is to making any product description that a customer can imagine, the more open the company’s workforce needs to be to the adaptation of production activities through novel task descriptions. However, at the same time, the company’s workforce needs to minimize manifest task entropy in carrying out production activities.

Iterations of negotiations with individual customers can lead to one-of-a-kind product descriptions, accompanying work descriptions and associated work schedules, which can lead to minimizing the manifest task entropy in planned production activities. However, many disturbances to planned production work can arise from the novelty of each one-of-a-kind product. This is because production can involve formed materials, parts, sub-assemblies, and assemblies that have characteristics and suppliers that ETO/MTO companies have not worked with before. Hence, ETO/MTO companies may not have developed working methods for exactly those components. Moreover, the supply of formed materials, parts, sub-assemblies, and assemblies can be delayed by the iterations between customer and company taking a long time. This can lead to many components being delivered in a short amount of time; short-term storage of unfamiliar components on the factory floor; and having to work around components on the factory floor. This can lead to overlapping disturbances to planned work from, for example, inadvertent blocking of process routes and increased potential for component breakages.

## 4. Automated Negotiation

As explained above, negotiation can be carried out in terms of product descriptions, product price, and production durations at the interface between the company and its customers in the business environment. Then, negotiation can be carried out in terms of workforce task descriptions derived from customer orders at the interface between the company’s sales department and the company’s production department. In this section, the relevance of negotiation protocols to balancing latent system entropy and manifest task entropy in workforces comprising humans and robots is explained. First, an overview of developments in automated negotiation over the past forty years is provided. Then, negotiation protocols are related to first principles.

### 4.1. Negotiation Protocols

Negotiation is a well-established topic in the intelligent systems literature. Already in the 1980s, negotiation was explained in terms of distributed problem solving by distributed artificial intelligence [58]. In the 1990s, it was argued that automated negotiation can be defined as “the process of information exchange by which the agents act to resolve inconsistent views and to reach agreement on how they should work together in order to cooperate effectively” and that “negotiation may be a recursive, complex, and pervasive process that is used to resolve conflicts in both domain-level and control-level problem solving” [59]. By this time, automated negotiation had become a topic of interest in production [60]. In the 2000s, it was opined that “perhaps the most fundamental and powerful mechanism for managing inter-agent dependencies at run-time is negotiation: the process by which a group of agents come to a mutually acceptable agreement on some matter. Negotiation underpins attempts to cooperate and co-ordinate (both between artificial and human agents) and is required both when agents are self-interested and when they are cooperative” [61]. Similarly, in the production literature, it was proposed that automated negotiation should encompass competition, cooperation, and their combination in so called, co-opetition [62]. In the 2010s, it was reported that automated negotiation was a common approach to making decisions and managing disputes between computational entities in order to achieve optimal agreements [63]. By this time, interest in automated negotiation in production had extended to multilateral negotiations in supply chain management [64]. In the 2020s, it has been reported that “automated negotiation has gained importance in the recent years owing to the growth in e-commerce and cloud-based applications. In a multi-agent environment, a negotiating agent exhibits autonomy and hence does not require a human during negotiation” [65]. Similarly, the sophistication of automated negotiation in intelligent production systems had increased [66]. In addition, there is now some interest in entropy-based automated negotiation for intelligent systems [67]. However, this has not extended to the practicalities of intelligent production. Today, as in the past, automated negotiation continues to involve negotiating agents dealing with their imperfect knowledge of each other through cycles of bidding and learning [68,69].

### 4.2. First Principles and Automated Negotiation

Within nature, agents and environments do not have perfect knowledge of each other. Nonetheless, back-and-forth reciprocal exchanges can minimize the entropy of information uncertainty, physical disorder, and energy loss between them. These exchanges can be considered to be oscillating interactions, which are needed to enable them to be in synchronous near equilibrium steady states. It is being in synchronous near equilibrium steady states that enables both to be able to adapt to each other while each maintains internal stability. Hence, although one side may be more stubborn than the other in the short-term, it is essential that both agents’ and environments’ near equilibrium steady states are mutually flexible over the longer-term in order to avoid so called oscillation death within which neither survives [70,71]. Similarly, automated negotiation deals with imperfect knowledge between agents through recursive information exchange [68,69]. Furthermore, automated negotiation can resolve inconsistent views and enable agreements about how to cooperate effectively [59]. Moreover, automated negotiation enables co-ordination and cooperation even though agents can be self-interested when seeking cooperation [61].

## 5. Mathematical Negotiation Model

In Section 6, we present an example of intelligent system control where production machines request tending services from robot and human agents. The overall goal is to keep the tardiness of the agents as low as possible, i.e., for the agents to carry out their tending tasks in due time as much as possible before the machines become idle.

### 5.1. Latent System Entropy

The range of possible requested tasks is represented by a set, *S*, of task labels for types of production services:

*S* = {*task*_1_, *task*_2_, …, *task_n_*} (1)

The manufacturing system for production services is composed of a set of production resources, i.e., in our case we consider human workers, autonomous mobile robots (AMRs), and a central coordinator as a set of agents, *A*:

*A* = {*agent*_1_, *agent*_2_, …, *agent_m_*} (2)

Each agent, *agent_i_*, has an associated set of tasks it can carry out, i.e., capabilities, *C_i_*, such that *C_i_* ⊆ *S*. Production jobs are released to the production control system as orders, *O_k_*, at time *k*, with each order specifying the type of task, the target for the service, and the due time [72]:

*O_k_* = {“*task_label*”: <*task_k_*>, “*target_label*”: <*target_k_*>, “*due_time*”: <*time_k_*>} (3)

Targets are classified for production tasks; here we consider the class “*location*”:

*T* = {*T*_1_, *T*_2_, …, *T_N_*}, *T_i_* ={“*target_label*”: <*target_k_*>, “*target_type*”: <*type*>} (4)

where *T* is the set of targets, which here is a location, and such locations are defined by a list of bounding coordinates:

<*location_k_*> = {(*x*_1_, *y*_1_), (*x*_2_, *y*_2_), …, (*x_n_*, *y_n_*)} (5)

which are assumed to compose a convex hull.

The goal for the production system is to be able to carry out an ordered servicing job with minimum tardiness of the agent, i.e., waiting (idle) times for the machines, with the states “waiting for parts”, “waiting for parts to be loaded”, and “waiting for parts to be unloaded” (here we assume that an order handling system has resolved the location from machine data, to be included into the order as “location”). The system should have an agent available as often as possible to carry out the requested task. For computing the latent “system” entropy, the probability of the system having the capability to carry out task *j* with probabilities for requested tasks is:

*P_j_* = *n_task_j_*/*n_all_tasks_* (6)

where *n_task_j_* is the number of agents capable of carrying out task *j* and *n_all_tasks_* is the number of all tasks that all the agents can carry out. Agents are fully independent from each other, and tasks are fully independent from each other. In particular, as is common in industrial practice, each individual machine tending task is carried out by one agent alone. As stated above in Section 4, competition, cooperation, and their combination in co-opetition can be facilitated through bidding in negotiation protocols. However, this does not require any dependencies between specific agents or between specific tasks. Rather, as explained in more detail below, specific agents are allocated specific tasks based on their specific independent availability, as represented by their separate bids. Thus, agents are in independent in co-opetition by independent bidding that is followed by accepting task allocation independently based on bid selection decisions.

The Boltzmann–Gibbs formulation of the latent system capacity, which encompasses in bits at time *t* both the true latent entropy and any information contents of the set of capable tasks, is defined as:

(7) $$H_{s}=-\sum_{j=1}^{n}P_{j,t}\;log2P_{j,t}\;$$

where *H_s_* is the latent system entropy considering the capability to carry out tasks at time *t* and *P_j,t_* is the probability of the system having the capability to carry out task *j* at time *t* [72].

### 5.2. Manifest Task Entropy

In machine tending, to keep the machine operations synchronized, it is important that the loading/unloading and transportation of parts and preforms can be done at the correct times. For recovering from disruptions such as the accidental blockage of pathways between the machines and between the machines and storage areas, it is critical to complete the tasks in due time. Task entropy is taken here as a measure for expected task quality, in particular, the potential capability for each independent agent to align with the requested time window for carrying out the requested task. At any particular time, it is possible that several agents or no agents may be available to carry out the requested task within the requested time window. If no agents are available, the request can be changed. Let *t_req_* be the machine’s requested starting time for the task and *t_bid_* be the starting time proposed by each potential tending agent, which may or may not make a bid within the requested time window.

**Figure 1:**
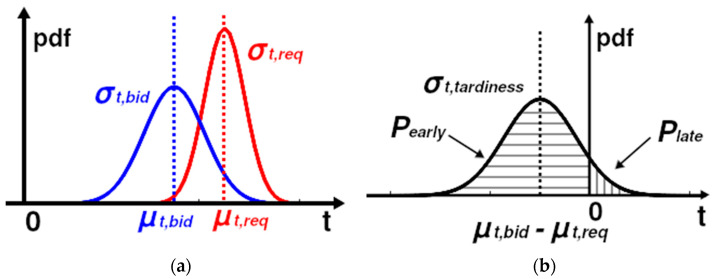
(**a**) Requested and bid starting times as Gaussian random variables; (**b**) Probabilities of being early and late based on the PDF of the tardiness.

In our simulated machine tending scenario, machines work autonomously for a fixed amount of time, after which they make a request to be serviced by an agent at a time in the future which has some uncertainty, represented by a Gaussian variable with mean *µ_req_* and standard deviation, *σ_req_*. After the service request is made, the machine continues working for this additional amount of time. Ideally, an agent will arrive before the service request time elapses, so servicing can begin immediately after the work ends. If no agent is present, however, the machine goes idle while waiting for an agent to arrive. Our goal then is to avoid tardiness of the servicing agents, which is defined as:

*t_tardiness_* = *t_bid_* − *t_req_* (8)

where *t_tardiness_* is the tardiness time. Let requested and bid starting times be Gaussian random variables, expressed with means and variances (or first standard deviations), also illustrated in Figure 1:

*t_req_* ~ *N*(*µ_req_*, *σ_req_*^2^) (9)

where *N* denotes the normal distribution, *µ_req_* is the mean of the requested service time, and *σ_req_* is the first standard deviation of the requested service time:

*t_bid_* ~ *N*(*µ_bid_*, *σ_bid_*^2^) (10)

where *µ_bid_* is the mean of bid service time and *σ_bid_* is the first standard deviation of the bid service time. Then, the tardiness becomes a Gaussian random variable:

*t_tardiness_* ~ *N*(*µ_tardiness_*, *σ_tardiness_*^2^) (11)

where *µ_tardiness_* is the mean of the requested service time and *σ_tardiness_* is the first standard deviation of the requested service time. The mean of the tardiness time is the difference:

*µ_tardiness_* = *µ_bid_* − *µ_req_* (12)

The first standard deviation of the tardiness time is the square root of the sum of variances of the requested and bid times:

*σ_tardiness_* = sqrt (*σ_req_*^2^ + *σ_bid_*^2^) (13)

The cumulative distribution function of the normal random variable is:

(14) $$\Phi x=\int_{-\infty }^{x}\varphi \left(t\right)dt$$

where $\Phi \left(x\right)$ is the cumulative distribution function and $\varphi \left(t\right)$ is the probability density function.

It may be noted that the cumulative distribution function cannot be expressed in a closed form [73]; however, numerical solutions and several approximations are available for it. We rely on libraries of the GNU Octave open-source scientific computing environment [74], namely, the *normcdf()* function of the Statistics package, to solve Equation (14) numerically. The probability of the bid being early is the cumulative distribution function of tardiness at *t* = 0, which is the same as the probability of the tardiness being negative:

(15) $$P_{\mathrm{early}}=\Phi_{\mathrm{tardiness}}(0)=\int_{-\infty }^{0}\varphi_{\mathrm{tardiness}}\;(t)$$

where *P_early_* is the probability of the bid being early, *Φ_tardiness_* is the cumulative distribution function of the tardiness, and *φ_tardiness_* is the probability density function of the tardiness, which depends on the mean and standard deviations of the two variables introduced above, i.e., Gaussian random variables: the requested service starting time, *t_req_*, and bid service starting time, *t_bid_*.

The tardiness (=service being late) is calculated as a sum of two Gaussian variables (requested service time by the machine and the estimated service time by the agent), with a resulting mean and standard deviation. The probability of the service of the agent being late is calculated from the probability distribution of this sum variable (see Equations (11)–(13)); the mean of the tardiness is not necessarily at zero, and the probability of being late is thus not constant. This is illustrated in Figure 1b above. The probability of the bid being late, *P_late_*, is simply:

*P_late_* = 1 − *P_early_* (16)

The task entropy is then estimated as:

(17) $$H_{t}=-P_{early}log2P_{early}-P_{late}log2P_{late}$$

where *H_t_* is the task entropy considering the capability of the agent to keep the requested schedule.

Maximizing the probability of the bid being early also minimizes the task entropy when the mean of the bid time is less than the mean of the requested service time. This is the desired case. However, when the mean of the bid time is more than the mean of the requested service time, then minimizing the task entropy would maximize tardiness. 

### 5.3. Negotiation Model

A negotiation protocol is applied to acquire the data to calculate the latent and task entropies in resolving which agent should be granted the order. We use an additional agent, a “coordinator” to control the communication with the robot and human agents, following the standard form of the well-known Contract Net protocol [75]. The coordinator requests a service from the agents, i.e., human operators and AMRs, to carry out a task. Here, we consider that the task specification must include the necessary information for calculating the entropy values, i.e., at least a task label and a start time. In practice, other information is also necessary, such as the target location and link (e.g., product ID) to the details of the handled parts and products. After the request, each agent replies with their capabilities (task labels) and a bid as the expected start time of the requested task.

The coordinator then calculates the latent system entropy and selects a system configuration (i.e., the set of remaining agents available after awarding the task) so that the latent system entropy is maximized for the case of a particular agent being granted the order. If there are several agent candidates, the selection of which results in the same level of system entropy, then such an agent is selected which bids the lowest task entropy, i.e., is expected to best keep with the requested schedules.

## 6. Simulations

In this section, the first principles analysis of system intelligence is related to everyday challenges in production systems through multiple simulations of the negotiation model.

### 6.1. Comparison of Task Allocation Methods

#### 6.1.1. Simulation Environment

**Figure 2:**
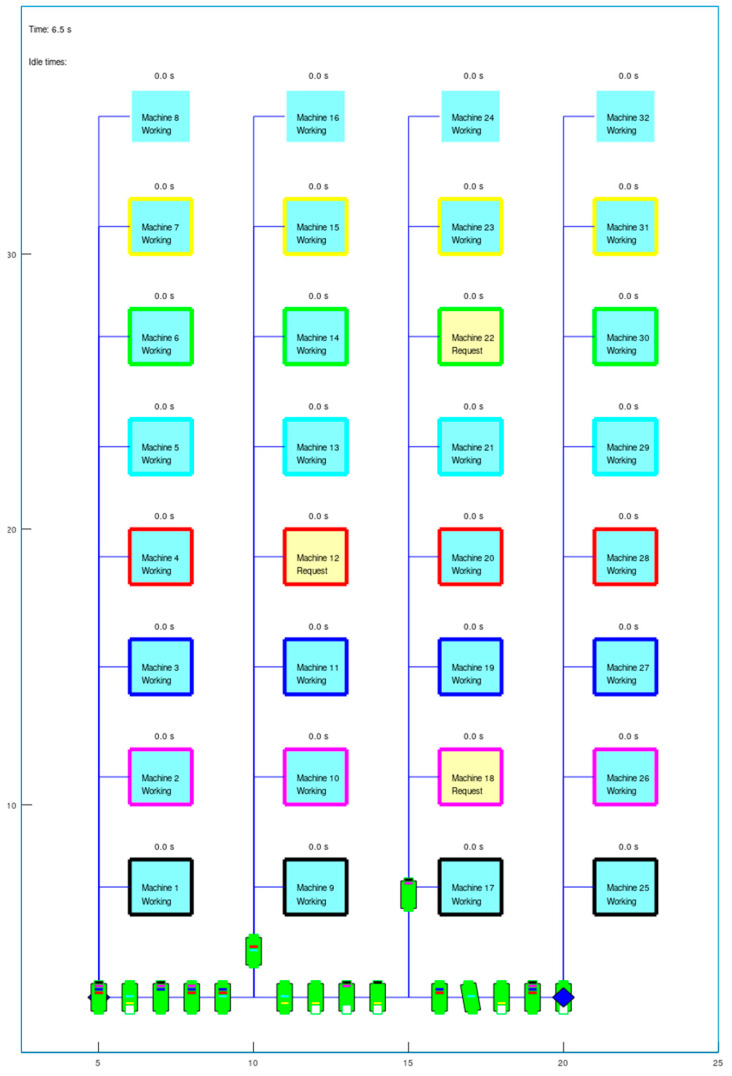
Layout of the shop floor simulation: four similar lines, eight different machines (marked by edge colour) in a line, and a set of agents (green rectangles—AMRs; blue rhomboids—humans) in their home positions (bottom line). Two agents are moving to tending positions.

To test the performance of entropy-based task allocation, a set of simulation tests was run in a discrete event simulator [76] implemented using Octave [74]. The simulated shop floor environment is a production facility consisting of eight different types of machines which are tended by AMRs or human operators. The machines are organized into four separate and identical lines, each consisting of eightmachines, one of each type (marked with different edge colours, see Figure 2).

#### 6.1.2. Agent Capabilities and Simulation Parameters

**Figure 3:**
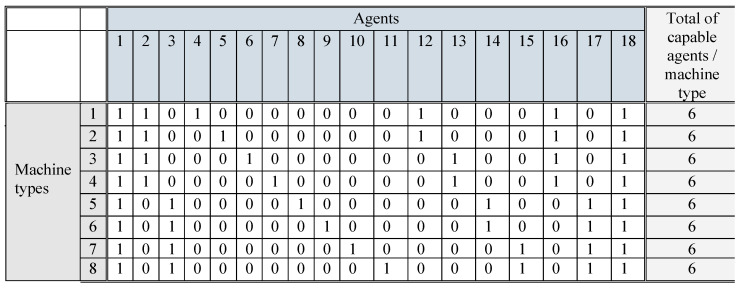
Capability matrix of the agents: human agents #1, #18, and AMR agents #2–#17.

There were two types of agents in the simulations: AMRs and human operators. Human agents could tend any type of machine, but their availability for machine tending was limited in time because their primary task was to carry out some manual production operations, such as packaging. AMRs had agent-specific capabilities of tending a selection of machine types. The capabilities were defined in a capability matrix where each column represented an agent and each row represented a machine type: ‘1’ for being capable of tending that machine type and ‘0’ for not being capable (see Figure 3).

A simulation begins with all agents at their home positions along the bottom lane: sixteen AMRs (green rectangles) and two humans (blue diamonds) (see Figure 2). One simplification in the simulator is that agents are able to drive through each other and occupy the same space, since the main purpose was to simulate approximate driving times and not develop collision avoidance strategies.

For AMRs, the home position serves as their charging station. The machines’ work cycle begins by working autonomously for 150 s, as most are doing in Figure 2 (filled with light blue colour). When this time elapses, they make a request for servicing, as Machines 12, 18, and 22 have done in Figure 2 (filled with yellow colour). After making a request, a machine continues working for an amount of time with a Gaussian variability (*request time* from Section 5.2: *µ_req_* = 50 s, *σ_req_* = 2 s) in order to introduce some uncertainty as to when exactly servicing needs to begin (discussed further in Section 6.1.3). In the meantime, an allocation process occurs where the servicing task is assigned to an agent if at least one is available and capable of servicing that type of machine. An agent is considered available if it is either waiting at its home position or driving back from a servicing job in one of the four main lanes or along the bottom lane.

**Figure 4:**
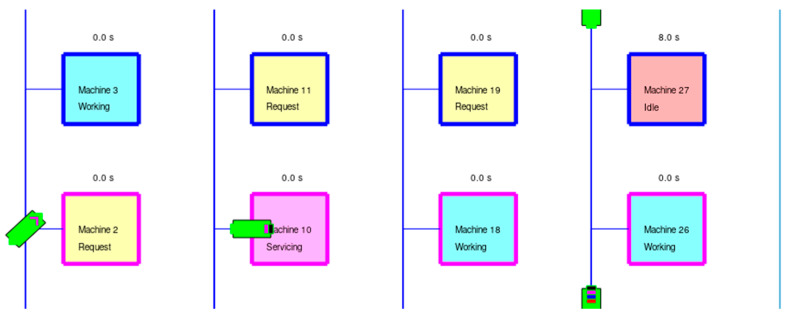
Part of the shop floor from Figure 2 later in the simulation. Machine 10 is being serviced by an AMR and Machine 27 has gone idle while waiting for servicing.

If the job is allocated, the agent goes to the machine and waits until it is finished working; then, servicing begins, which lasts 20 s (see Machine 10 in Figure 4). With servicing complete, the machine begins a new work cycle, and the agent returns to their designated home position. If no agent is present when the work period is finished, the machine goes idle (see Machine 27 in Figure 4) and waits for an agent. One goal in the simulations and allocation strategies presented here is to reduce this machine idle time.

#### 6.1.3. Uncertainties in the Servicing Timeliness

Some additional uncertainty was introduced to the simulation model by adding Gaussian variability to the agents’ bids in the allocation process. This is the *bid time* (*µ_bid_*, *σ_bid_*) in Section 5.2, i.e., the time agents say they are ready to tend the requesting machine, and is used to calculate the probability of the agents arriving early together with the machine *request time*. The bid time is then also used to add some randomness to the agents’ realized servicing times.

The bid time (*µ_bid_*, *σ_bid_*) is generated for each agent for each allocation from their maximal and mean driving speed and the driving path lengths. The maximum agent speed, *v_max_*, was set at 1 m/s, while the mean speed, *v_mean_*, varied linearly from 0.7 m/s to 0.9 m/s, depending on the driving distance, *d_path_*, from the agent’s location to the machine making the request. The slowest mean speed corresponds to the longest driving path from a home position to a machine, while the fastest speed corresponds to the shortest such path. The reasoning behind this is that for longer paths, there would be a higher chance of encountering an obstacle or traffic, requiring extra time for maneuvering (though obstacle avoidance was not actually part of the simulation).

The AMRs are simulated as being differential drive robots which drive straight path segments and then turn on the spot; however, the turning time is neglected here when determining *µ_bid_*. Once *v_mean_* is determined, *µ_bid_* is calculated:

*µ_bid_* = *d_path_*/*v_mean_* (18)

To determine *σ_bid_*, the nominal driving time, *t_nom_*, at *v_max_* is first calculated:

*t_nom_* = *d_path_*/*v_max_* (19)

The difference between the nominal and mean driving time is defined as three times the first standard deviation, thus:

*σ_bid_* = (*t_nom_* − *µ_bid_*)/3 (20)

Once an agent is allocated a task, the actual driving time, *t_drive_*, is randomly generated with a Gaussian distribution from the bid time (*µ_bid_*, *σ_bid_*) using Octave’s [73] randn() function:

*t_drive_* = *µ_bid_* + randn() × *σ_bid_* (21)

The driving speed, *v_drive_*, to use in the simulator for that allocated job is then finally calculated:

*v_drive_* = *d_path_*/*t_drive_* (22)

The human agents are given a ten times larger timing uncertainty, *σ_bid_*, compared with the AMR agents. This is because the human agents are considered not to be working primarily on the machine tending tasks, but on tasks such as packaging or assembly, with machine tending being a secondary task for them. As the human worker must prioritize ongoing tasks not related to machine tending, the uncertainty of their arrival time at the machine to be tended is much higher than that of the AMR. When computing a human agent’s actual speed, *v_drive_*, using Equations (21) and (22), the higher value of *σ_bid_* sometimes results in *v_drive_* being higher than *v_max_* (1 m/s), though it appeared to occur rarely. It also was assumed to have a small effect on the results, as there were only two human agents and sixteen AMRs.

#### 6.1.4. Initial Conditions for the Machine Tending Requests

At the start of a simulation, the machines are initialized with a random elapsed working time, which is a fraction of the initial 150 s of working time before they make a service request. This “initial request value” was defined separately for each machine. In this way the first tending requests were distributed in time instead of having them in time clusters. The initial request value was a uniformly distributed random float number ranging from 0 to 1, defining the time of the first request the machine makes depending on the working time of the machine (machWorkTime = 150 s):

firstRequest = machWorkTime × initialRequestValue(23)

**Figure 5:**
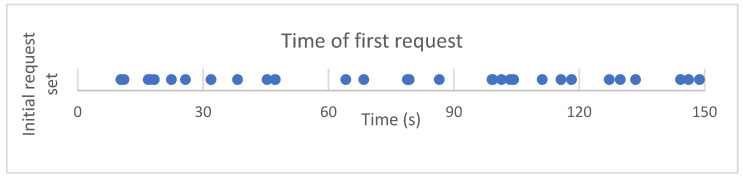
Machines’ first requests plotted on a timeline.

The same initial timing set was used in all of the simulations. Figure 5 displays the first requests in a timeline and shows that they come in relatively evenly during the 150 s machine worktime window.

#### 6.1.5. Task Allocation Methods

Five different task allocation methods were tested with the simulations, from which details are given in Table 1. The methods HIGHPROB, MINCAP, TIMEDIFF, and CLOSEST have a linear computational complexity (O(n)), as they only need to iterate through the agents once while comparing their bid values. The SYSENT method is more computationally expensive (quadratic, O(n^2^)); it iterates through all the agents, but to calculate the system entropy associated with selecting each agent, it must iterate again through all the remaining agents to compute their task capabilities.

The navigation time from an available AMR’s current position to a machine location is estimated and used to offer a servicing bid using the HIGHPROB allocation method, i.e., the probability of arriving early. As described in Section 6.1.3, this time is based on the driving distance and driving speed and is a random value with a normal distribution. In simulation tests, the actual driving speed used for a driven path, regardless of the allocation method, is randomly generated from this normal distribution. The navigation time directly affects the task allocation method when the closest distance is used as the task allocation criterion (CLOSEST), favouring agents with short distances to the target. Indirectly, it also affects other task allocation methods where the probability of being late is considered as the task allocation criterion, either as a direct criterion (HIGHPROB) or as a tiebreaker (SYSENT, MINCAP). If the distance from the agent to the target is large, it may not arrive early enough at the target (the CLOSEST criterion). In addition, the probability of the agent getting late to the target may get higher because the mean of the expected time may be close to the requested time, or even later than the requested service time (HIGHPROB, TIMEDIFF).

#### 6.1.6. Task Allocation Goals

The desired performance for the machine tending agents is one in which the tending service can be offered in a timely manner, i.e., the agent must arrive at the desired machine before the machine goes idle. However, we do not want the agent to arrive at the machine too early, as then the agent would be idling at the machine, waiting for it to be ready to be tended. Therefore, the tardiness of the agents must be minimized without adding significant waiting time at the machine.

In addition to minimizing idling time of agents at the machines, we must ensure some idling time for the agents at their home position. This would allow them to charge their batteries between tending tasks (AMRs) or do their primary work (human agents). Consequently, we are on the lookout for an allocation method which offers us reasonably low tardiness values, minimal idling at the machine (timely servicing), and enough idling time at the home position to charge batteries or do primary work.

### 6.2. Simulation Results

Simulation runs were done with the settings and parameters explained above for 2000 s of simulated time. The parameters given in Table 2 were tracked during the simulation to follow the performance of the different allocation methods. 

In the simulation tests, the mean values for each allocation method of *allocSysEnt*, *tardiness*, *numIdle*, and *numAvailable*, as well as the cumulative *idlingTime* and *availableTime* were recorded and are shown in Table 3.

From the mean values we can see that with these simulation settings, using the SYSENT allocation method provides the least tardiness and highest cumulative idling time for the agents. A lower tardiness corresponds to a higher probability of arriving early and therefore also a lower task entropy (see Equation (17), Section 5.2). When the agents are idling more with the SYSENT method, this would translate to increased charging times with an actual mobile robot or time to be used in the primary work for human operators. Additionally, the availability time is highest and the number of available agents is highest using SYSENT.

**Figure 6:**
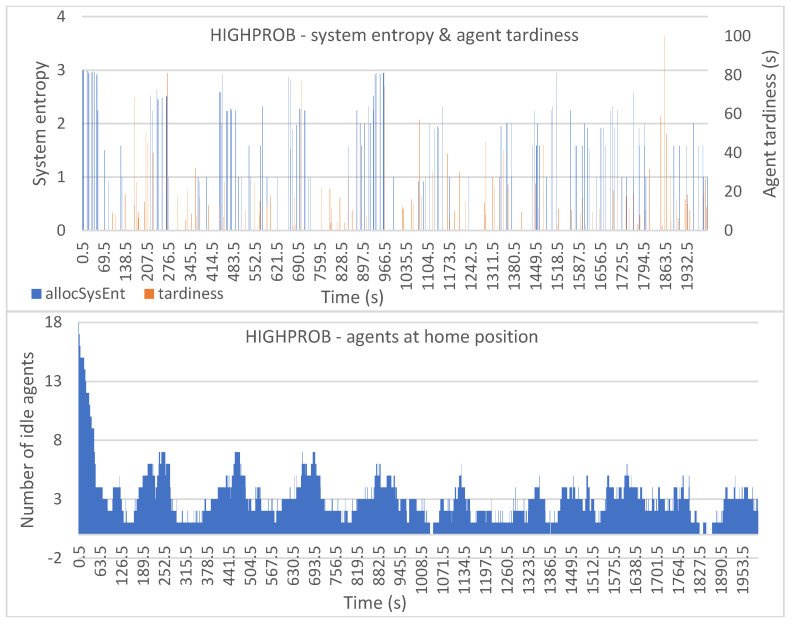
Simulation results with the HIGHPROB allocation method: system entropy and agent tardiness (**top**); number of idle agents (**bottom**).

**Figure 7:**
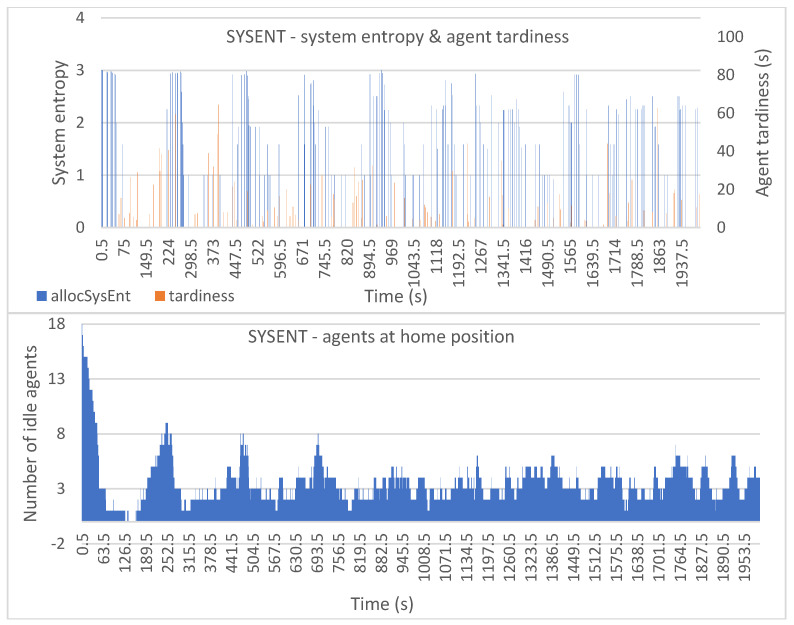
Simulation results with the SYSENT allocation method: system entropy and agent tardiness (**top**); number of idle agents (**bottom**).

**Figure 8:**
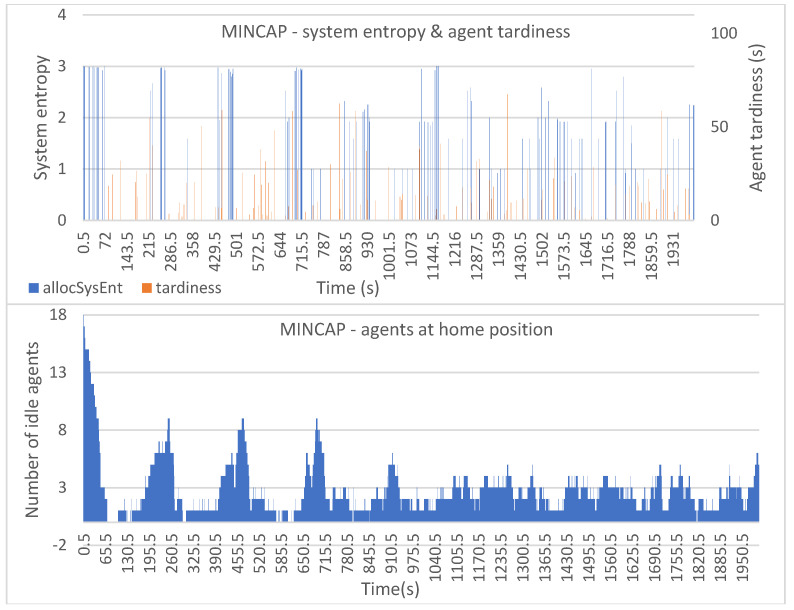
Simulation results with the MINCAP allocation method: system entropy and agent tardiness (**top**); number of idle agents (**bottom**).

**Figure 9:**
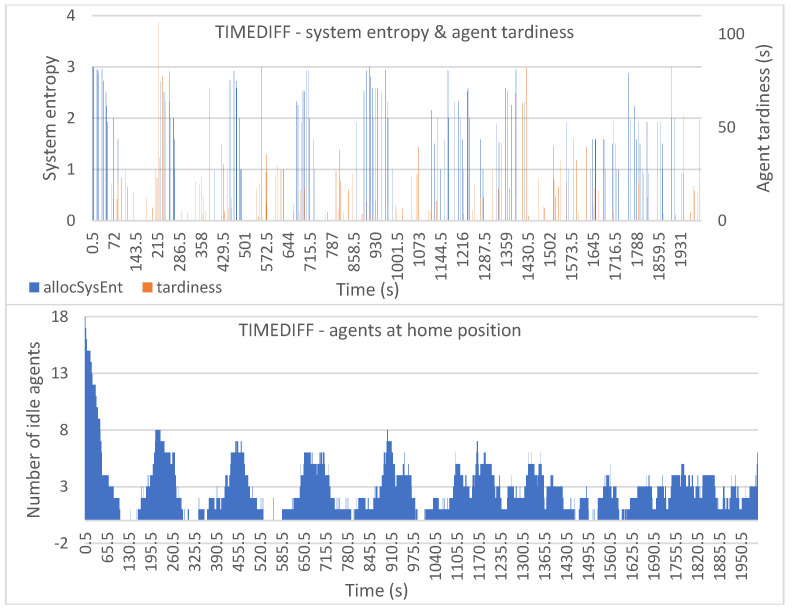
Simulation results with the TIMEDIFF allocation method: system entropy and agent tardiness (**top**); number of idle agents (**bottom**).

**Figure 10:**
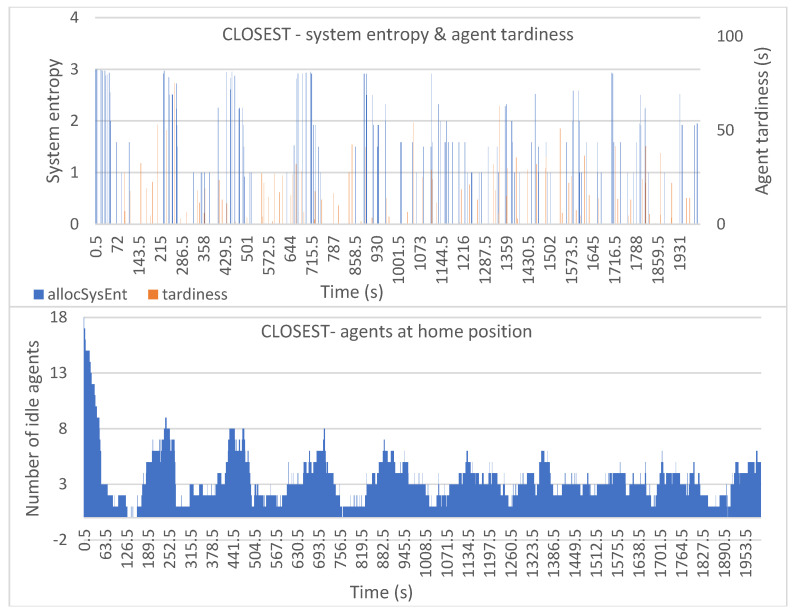
Simulation results with the CLOSEST allocation method: system entropy and agent tardiness (**top**); number of idle agents (**bottom**).

Figure 6, Figure 7, Figure 8, Figure 9 and Figure 10 show the plotted data for *allocSysEnt* and service tardiness (top plots) for each allocation method. On the X-axis, we have the simulation time, and on the left Y-axis (top plots), we have the realized system entropy after an allocation. On the right Y-axis (top plots), we have the agent tardiness value. System entropies are plotted after each allocation and tardiness is plotted every time an agent reaches a machine to be tended. With each allocation method, it can be seen that there are more cases of agents arriving late when the system entropy is very low (=zero in many cases). Figure 6, Figure 7, Figure 8, Figure 9 and Figure 10 (bottom plots) also show data for the number of idling agents at a given time during the simulation.

The HIGHPROB allocation method provides us with relatively even tardiness of agents throughout the simulation. In Figure 6 (top), we can see some high peaks in the tardiness values and the system entropy fluctuating. Figure 6 (bottom) displays that with this allocation method there is at least one agent idling at almost all times. 

Predictably, the SYSENT allocation method keeps the system entropy higher than other allocation methods during the simulation. Figure 7 (top) shows that some tardiness is present during the entire simulation time, but the tardiness values are very low with no present peaks. The agent tardiness seems to have higher values when the system entropy is on the low end. From Figure 7 (bottom) we can see that this allocation method allows the AMRs to idle. Only in the beginning of the simulation are there no idling AMRs, and for the rest of the simulation that number rarely goes under two. 

In Figure 8 (top) we can see that the MINCAP allocation method gives us slightly more fluctuating system entropy values with slightly higher agent tardiness values without very high peaks in the tardiness. It seems that with this method the agents arrive quite consistently late without bigger delays (such as the delays that we see with HIGHPROB in Figure 6 and TIMEDIFF in Figure 9), but as the method selects the least capable agents for the tasks, the group of agents that gets left tends to be highly capable, resulting in the system tending to be prepared for many types of tasks. This might explain why there is no high tardiness values, as the system is more likely to have a capable agent idling. 

With the TIMEDIFF allocation method (Figure 9 (top)) we can see highly fluctuating system entropy values and tardiness during the whole simulation time. Tardiness values have some high peaks which might be because the allocation method does nothing to consider the capabilities of the agents, so there is a higher chance of an allocation leaving behind a less capable agent group. This can result in no capable agents being available for a tending request and the requesting machine waiting for a previous tending mission to finish to get a capable agent to serve the machine. From Figure 9 (bottom) we can also see that the number of idling AMRs is noticeably lower than it is with other allocation methods. 

In Figure 10 we can see relatively low tardiness values with no major peaks but some still notably high tardiness values. This too might be because of the at times low system entropy; if there is no capable agent in the group of available agents, there will be high values in the lateness of the agents.

## 7. Conclusions

### 7.1. Principal Contributions

The paper provides four principal contributions. First, system intelligence is explained in fundamental terms. Specifically as capability to maintain internal stability while adapting to changing environments through minimizing manifest task entropy and maximizing latent system entropy. Second, how automated negotiation relates to balancing the stability of minimum manifest task entropy and the adaptability of maximum latent system entropy has been explained. Third, a mathematical negotiation model has been presented that enables the balancing of manifest task entropy and latent system entropy in intelligent systems. Fourth, this first principles analysis of system intelligence has been related to everyday challenges in production systems through multiple simulations. The simulations indicate that manifest task entropy, in particular uncertainty about the time that will be taken to carry out a machine tending task, is minimized when maximizing latent system entropy is used as the criterion for task allocation. Overall, the paper goes beyond previous literature that is concerned with relating insights from nature to intelligent production [14,15,16,17,18,19,20,21,22], but which has not addressed interplay between adaptability and stability in terms of latent system entropy and manifest task entropy. In particular, this paper introduces a new perspective of how first principles can be applied in systems that encompass natural and artificial intelligence. This new perspective goes beyond the state-of-the-art formulations based on first principles and entropy dynamics [23,24,25,26].

### 7.2. Practical Implications

As balancing maximum latent system entropy for adaptability and minimum manifest task entropy for stability is fundamental to survival in changing environments, there are many relevant practical situations. Consider, for example, the following headline of an article in Forbes Magazine: “Covid Shortages: Supply chains must become less efficient”. In that business magazine article, it is reported that “companies are looking at more flexible operations and resilient supply chains, even if means sacrificing some profitability in the short-term” [77]. Changing environments can range from sudden abrupt changes in entire natural landscapes because of extreme weather events to unpredictable changes in retail fashion markets. Abrupt changes to natural landscapes can cause disruptions in agri-food production. Without maximum latent production system entropy and minimum manifest production task entropy, for example, through dynamic organization of moveable factories [78], it may not be possible to maintain agri-food production at affordable costs. The timing of changes in retail fashion markets can be predictable if they are based on regular seasonal collection launches related to fashion weeks in Paris, Milan, etc. However, markets can change because of less predictable variables such as social media responses to launches. Accordingly, brands need to be able to change sources and configurations of thousands of different components from global sources in order to be able adapt with changing fashion markets while maintaining affordable production costs [79].

### 7.3. Directions for Future Research

A major research question is how can first principles be operationalized to improve the performance of intelligent systems. Albert Einstein opined that: “As far as the laws of mathematics refer to reality, they are not certain; and as far as they are certain, they do not refer to reality” [80]. Accordingly, rather than basing intelligent systems on natural sciences’ correspondence truths, it may be more productive to base them on engineering sciences’ pragmatic truths: i.e., the truth is what works [81]. For example, instead of trying to develop intelligent systems from whatever mathematical statements are considered currently to correspond exactly with nature’s first principles, it may be more productive to base them on the application of established computational methods, such as automated negotiation, which are congruent with first principles but which have emerged without deliberative reference to first principles. Hence, future research could compare correspondence truths and pragmatic truths as different bases for the development of intelligent systems.

## Figures and Tables

**Table 1:** Task allocation methods.

Allocation Method	Description
HIGHPROB	Highest probability of arriving early. The job gets allocated to the AMR which has the highest probability of arriving early at the machine to be tended. SYSENT is used as a tiebreaker.
SYSENT	Maximizes system entropy: The job gets allocated to the AMR whose selection maximizes the system entropy of the remaining available agents. HIGHPROB is used as a tiebreaker: if two or more agents give the same system entropy, then the one with the best HIGHPROB gets the order.
MINCAP	Minimal capabilities: The job gets allocated to the AMR with the fewest capabilities. HIGHPROB is used as a tiebreaker.
TIMEDIFF	Minimum time difference between the request and the bid (abs. value): The agent should arrive at the machine as close to the service time as possible.
CLOSEST	The job gets allocated to the capable agent closest to the requesting machine.

**Table 2:** Parameters recorded during simulations.

Parameter	Description	Recording
maxSysEnt	Maximum possible system entropy after a possible allocation. Not necessarily the realized system entropy, as the allocation method does not necessarily maximize the system entropy.	Value saved after each allocation
minSysEnt	Minimum possible system entropy after a possible allocation. Not necessarily the realized system entropy, as the allocation method does not necessarily minimize the system entropy.	Value saved after each allocation
allocSysEnt	The actual system entropy after an allocation.	Value saved after each allocation
Tardiness	The tardiness value (seconds) if the agent arrives late for a machine tending job.	Value saved each time an agent reached the machine to be served
idlingTime	Cumulative idling time of all agents. Idling is when the agent is in the home position in WAIT state.	Saved in each simulation loop iteration
availableTime	Cumulative time available among all agents. Agents are available while they are in the home position in WAIT state or when they are returning from a previous job and not yet allocated to a new job.	Saved in each simulation loop iteration
numIdling	Number of agents idling at a given time.	Saved in each simulation loop iteration
numAvailable	Number of agents available at a given time.	Saved in each simulation loop iteration

**Table 3:** Simulation results.

Allocation Method	Mean allocSysEnt (s)	Mean Tardiness (s)	Cumulative idlingTime (s)	Cumulative availableTime (s)	Mean numIdle (s)	Mean numAvailable (s)
HIGHPROB	1.56	7.94	6092	16255	3.05	8.13
SYSENT	1.89	5.79	6922	16397	3.46	8.20
MINCAP	1.65	7.84	5427	16123	2.71	8.06
TIMEDIFF	1.49	8.28	5706	16152	2.85	8.07
CLOSEST	1.69	6.91	6853	16324	3.43	8.16

## Data Availability

Data available on request to E.H.
